# Efficacy and Safety of Asparagusic Acid against *Echinococcus multilocularis* In Vitro and in a Murine Infection Model

**DOI:** 10.3390/tropicalmed9050110

**Published:** 2024-05-11

**Authors:** Zhuanhong Lu, Yating Wang, Chuanchuan Liu, Haining Fan

**Affiliations:** 1 School of Clinical Medicine, Qinghai University Affiliated Hospital, Xining 810001, China; ys221002100824@qhu.edu.cn (Z.L.); ys221001040783@qhu.edu.cn (Y.W.); 2 Qinghai Key Laboratory of Echinococcosis Research, Xining 810001, China

**Keywords:** alveolar echinococcosis, *Echinococcus multilocularis*, asparagusic acid, apoptosis, PI3K/Akt signaling pathway

## Abstract

Alveolar echinococcosis (AE) stands as a perilous zoonotic affliction caused by the larvae of *Echinococcus multilocularis*. There is an imperative need to explore novel therapeutic agents or lead compounds for the treatment of AE. Asparagusic acid, characterized by its low toxicity and possessing antimicrobial, antioxidant, and anti-parasitic attributes, emerges as a promising candidate. The aim of this study was to investigate the in vivo and in vitro efficacy of asparagusic acid against *E. multilocularis*. Morphological observations, scanning electron microscopy, ROS assays, mitochondrial membrane potential assays, and Western blot were used to evaluate the in vitro effects of asparagusic acid on protoscoleces. The effects of asparagusic acid on vesicles were assessed via PGI release, γ-GGT release, and transmission electron microscopy observations. CellTiter-Glo assays, Caspase3 activity assays, flow cytometry, and Western blot were used for an evaluation of the effect of asparaginic acid on the proliferation and apoptosis of germinal cells. The in vivo efficacy of asparagusic acid was evaluated in a murine AE model. Asparagusic acid exhibited a pronounced killing effect on the protoscoleces post-treatment. Following an intervention with asparagusic acid, there was an increase in ROS levels and a decline in mitochondrial membrane potential in the protoscolex. Moreover, asparagusic acid treatment resulted in the upregulation of PGI and γ-GGT release in metacestode vesicles, concomitant with the inhibition of germinal cell viability. Furthermore, asparagusic acid led to an enhanced relative expression of Caspase3 in the culture supernatant of both the protoscoleces and germinal cells, accompanied by an increase in the proportion of apoptotic germinal cells. Notably, asparagusic acid induced an augmentation in Bax and Caspase3 protein expression while reducing Bcl2 protein expression in both the protoscoleces and germinal cells. In vitro cytotoxicity assessments demonstrated the low toxicity of asparagusic acid towards normal human hepatocytes and HFF cells. Additionally, in vivo experiments revealed that asparagusic acid administration at doses of 10 mg/kg and 40 mg/kg significantly reduced metacestode wet weight. A histopathological analysis displayed the disruption of the germinal layer structure within lesions post-asparagusic acid treatment, alongside the preservation of laminated layer structures. Transmission electron microscopy further revealed mitochondrial swelling and heightened cell necrosis subsequent to the asparagusic acid treatment. Furthermore, asparagusic acid promoted Caspase3 and Bax protein expression while decreasing Bcl2 protein expression in perilesional tissues. Subsequently, it inhibited the expression of Ki67, MMP2, and MMP9 proteins in the perilesional tissues and curbed the activation of the PI3K/Akt signaling pathway within the lesion-host microenvironmental tissues. Asparagusic acid demonstrated a pronounced killing effect on *E. multilocularis*, suggesting its potential as a promising therapeutic agent for the management of AE.

## 1. Introduction

Alveolar echinococcosis (AE) is a severe chronic parasitic disease caused by the larval stage of *Echinococcus multilocularis*, resembling slow-growing liver cancer [1]. The disease exhibits a global distribution, with its highest prevalence recorded in the northern hemisphere, notably in China, where it accounts for 91% of cases [2], with some regions reporting human prevalence as high as 3% [3]. While AE predominantly affects the liver, it can metastasize to distant organs, such as the lungs, brain, kidneys, and bones, as well as peripheral tissues, such as the gallbladder, blood vessels, and biliary system [4]. Without timely intervention, the mortality rate of AE exceeds 90% within 10–15 years [5]. Surgery currently stands as the most effective treatment for AE. However, many patients miss out on this option due to the disease’s prolonged incubation period, lack of early symptoms, and metastatic progression [6]. Benzimidazoles, primarily albendazole, serve as first-line drugs for AE treatment. Nonetheless, albendazole’s limited absorption in the gastrointestinal tract and low bioavailability lead to inadequate drug concentrations at the lesion site, thereby curtailing its therapeutic efficacy [7]. Additionally, albendazole exerts a parasitostatic rather than a parasitocidal effect [8], necessitating prolonged administration to enhance therapeutic outcomes, albeit at the expense of increased risk of adverse reactions [9]. Consequently, there exists an urgent imperative to identify novel therapeutic agents or lead compounds to optimize AE treatment strategies.

Asparagus, a genus within the lily family, exhibits significant biological effects, including antimicrobial, antioxidant, and cytotoxic properties [10]. Notably, asparagus extract demonstrates potent cytotoxic activity against colorectal cancer [11]. Asparagusic acid (AA) is a sulfur-containing compound isolated from asparagus, and this component appears to be unique to asparagus [12]. While its precise biological function remains uncertain, studies suggest its efficacy in inhibiting fungal growth and repelling insects [13]. Moreover, asparagusic acid shows promise in treating wireworm infections [14]. This study aimed to assess the in vitro parasiticidal effects of asparagusic acid on protoscoleces, metacestode vesicles, and germinal cells, as well as its role in experimentally infected mice in vivo.

## 2. Methods

### 2.1. Animal and Ethical Statement

SPF-grade female C57BL/6J mice (18–20 g) were procured from Beijing HFK Bioscience Company and housed in individually ventilated cages within our laboratory facility. The ambient temperature was maintained between 21 °C and 23 °C, with the relative air humidity ranging from 45% to 55%. The mice were provided ad libitum access to food and water throughout the experimental period. Ethical clearance for the animal experiments was obtained from the Ethics Committee of the Qinghai University Affiliated Hospital (approval number: P-SL-2023-332). All procedures were conducted under 1% pentobarbital sodium anesthesia, paying meticulous attention to minimizing animal pain.

### 2.2. Cell and Chemical Reagents

Rat hepatoma (RH) cells, normal human hepatocytes, and human foreskin fibroblasts (HFFs) cells were procured from Procell (Wuhan, China). Asparagusic acid was obtained from MedChemExpress (Monmouth Junction, NJ, USA).

### 2.3. Separation of E. multilocularis Protoscoleces

*E. multilocularis* protoscoleces were derived from Mongolian gerbils maintained for seed preservation in our laboratory and confirmed as the *E. multilocularis* source via PCR [15]. The gerbils were humanely euthanized using cervical dislocation following anesthesia induction with 1% sodium pentobarbital. Subsequently, abdominal lesions were aseptically dissected within a biosafety cabinet. The excised lesion tissues were sectioned in pre-cooled phosphate-buffered saline (PBS), and the protoscoleces were sieved through four layers of sterile gauze into a 50 mL centrifuge tube. A 40 μm cell strainer was then employed to filter the protoscolex solution and eliminate calcium particles. The protoscoleces were subsequently subjected to ten washes with normal saline. Following the natural sedimentation of the protoscoleces, Roswell Park Memorial Institute 1640 (RPMI-1640) culture medium was added for resuspension, and the protoscolex concentration was adjusted to 1000/mL. The cultivated protoscoleces were cultured at 37 °C in a 5% CO_2_ incubator (HERAcell 150i, ThermoFisher Scientific, Waltham, MA, USA).

### 2.4. In Vitro Culture of Metacestode Vesicles

Metacestode vesicle culture materials were harvested from C57BL/6J mice infected via an intraperitoneal injection of 2000 protoscoleces, and the mice were subsequently housed for a 3-month period post-infection. Following euthanasia by an intraperitoneal injection of pentobarbital sodium and cervical dislocation, the mice were briefly immersed in 75% ethanol for 3 min. Subsequently, lesion tissues within the abdominal cavity were meticulously dissected under biosafety cabinet conditions (ESCO Lifesciences, Changi, Singapore). These lesion tissues were then minced and passed through a tea strainer (Migros, Berne, Switzerland) to obtain vesicle culture material. The acquired vesicle culture materials underwent 10 washes with normal saline, followed by resuspension in Dulbecco’s Modified Eagle Medium (DMEM) supplemented with 20% fetal bovine serum (FBS, Procell, Wuhan, China). Co-cultivation with 5 × 10^5^ RH cells was conducted in a 37 °C incubator with 5% CO_2_. The RH feeder cells and culture medium were refreshed every 4 days. Upon reaching a diameter of 2–4 mm, the vesicles were transferred to feeder cell-free culture flasks and placed in a sealed bag filled with nitrogen at 37 °C for one week to eliminate feeder cell contamination.

### 2.5. RT-PCR Identification of the Metacestode Vesicles and Germinal Cells

The total RNA was extracted from the metacestode vesicles and mouse liver tissue using an RNAsimple Total RNA Kit (Tiangen Biotech, Beijing, China) according to the manufacturer’s instructions. Subsequently, the RNA was reverse-transcribed into cDNA utilizing a FastKing gDNA Dispelling RT SuperMix Kit (Tiangen Biotech).

The presence or absence of host cell contamination in metacestode vesicles and germinal cells was determined using *E. multiloculus* GAPDH and mouse GAPDH as indicators. Specific primers were synthesized by Shanghai Sangon Biotech with the following sequences: EmGAPDH, forward: 5′-TTTCGCAGCCGATGCCCGAT-3′, reverse: 5′-CACCGCTGTGAGAGCAGCCA-3; mouse GAPDH, forward: 5′-CCACCATGGAGAAGGCCGGG-3′, reverse: 5′-ATGTTCTGGGCAGCCCCACG-3. The RT-PCR reaction was performed using a Mastercycler Nexus PCR apparatus to a final volume of 50 μL: 10 μL 2 × Taq PCR MasterMix, 0.6 μL (10 μM) for each primer, 2 μL cDNA, 6.8 μL ddH_2_O. Amplification was carried out under the following conditions: initial denaturation at 94 °C for 3 min; followed by 30 cycles of denaturation at 94 °C for 30 s, annealing at 60 °C for 30 s, and extension at 72 °C for 30 s; with a final extension at 72 °C for 3 min. PCR products were separated via 2% agarose gel electrophoresis and visualized under UV light after staining with GeneRed (Tiangen Biotech).

### 2.6. Effect of Asparagusic Acid on E. multilocularis Protoscoleces In Vitro

Next, 3 mL of protoscolex solution was added to a 6-well cell culture plate, and 2 mL of different amounts of asparagusic acid was added to make the final concentration of 0, 2.5, 5, 10, 20, 40, 80, 160, and 320 μM. The protoscoleces were cultured continuously for seven days in a 37 °C 5% CO_2_ incubator, and 0.5 mL of protoscolex suspension was aspirated daily to count protoscolex survival. Following the natural settlement of the protoscoleces, the culture medium was aspirated, and 15 μL of 0.1% eosin solution was added for staining. The stained protoscoleces were subsequently examined under a Zeiss microscope (Oberkochen, Germany), with dead protoscoleces appearing red due to eosin staining. Each concentration was subjected to three independent biological replicates. Additionally, a subset of protoscoleces was fixed in 2.5% glutaraldehyde buffer (Solarbio, Beijing, China) for an electron microscopy analysis.

### 2.7. Effects of Asparagusic Acid on Reactive Oxygen Species in E. multilocularis Protoscoleces

A Reactive Oxygen Species Assay Kit (Beyotime, Shanghai, China) was employed to assess the levels of reactive oxygen species (ROS) in the protoscoleces following an intervention with asparagusic acid (10, 20, 40 μM), adhering to the manufacturer’s protocol. In brief, the protoscoleces were treated with asparagusic acid for 48 h, followed by collection. Subsequently, the protoscoleces were incubated with a final concentration of 10 μM DCFH-DA for 30 min at 37 °C, shielded from light. After rinsing with RPMI-1640 culture medium, the protoscoleces were visualized using a Zeiss fluorescence microscope. ImageJ software (version 1.51J8, NIH, Bethesda, MD, USA) was used to calculate the mean fluorescence intensity.

### 2.8. Mitochondrial Membrane Potential Measurement

A Mitochondrial Membrane Potential Assay Kit (Rhodamine 123, Beyotime) was employed to evaluate the mitochondrial membrane potential of the protoscoleces post-intervention with asparagusic acid. Subsequent to asparagusic acid treatment, the protoscoleces were gathered in EP tubes, and after settling naturally, the culture supernatant was removed. Subsequently, the protoscoleces were incubated with 1 mL of Rhodamine 123 working solution at 37 °C for 30 min. Following the incubation period, the protoscoleces underwent two washes with pre-warmed RPMI-1640 culture solution at 37 °C. Finally, the protoscoleces were suspended in RPMI-1640 culture medium and mounted onto slides for examination using a Zeiss fluorescence microscope. ImageJ software was used to calculate the mean fluorescence intensity.

### 2.9. Alkaline Phosphatase (ALP) Activity Measurement

The ALP levels in the culture supernatant of the protoscoleces after asparagusic acid intervention were determined using an ALP assay kit (Elabscience, Wuhan, China) according to the manufacturer’s instructions. After the protoscoleces underwent the intervention with different concentrations of asparagusic acid for 48 h, the culture supernatant was collected and centrifuged at 10,000× *g* for 10 min at 4 °C, and the supernatant was taken for ALP determination.

### 2.10. Caspase3 Activity Assay

A Caspase3 activity assay kit (Elabscience) was employed following the manufacturer’s protocol to evaluate Caspase3 activity in both the protoscoleces and culture supernatant. Following treatment with asparagusic acid for 48 h at each concentration level, the protoscoleces were treated with 200 μL of pre-cooled PBS buffer. Subsequently, the supernatant was obtained by centrifuging the homogenized samples at 10,000× *g* for 10 min. The protein sample concentration in the supernatant was determined using the Bradford method. The supernatant was then incubated with Ac-DEVD-pNA (2 mM) at 37 °C for 2 h. Following incubation, the absorbance at 405 nm was measured using a spectrophotometer, and enzyme activity was calculated accordingly.

### 2.11. Metacestode Vesicle PGI and γ-GGT Release Assay

The phosphoglucose isomerase (PGI, Abcam, Cambridge, UK) method and gamma-Glutamyltransferase (γ-GGT, Nanjing Jiancheng Bioengineering Institude, Nanjing, China) assay were employed to quantify the release levels of PGI and γ-GGT in the culture medium of the metacestode vesicles following the intervention with asparagusic acid, serving as an indirect measure of the damage inflicted on the metacestode vesicles by the acid. Metacestode vesicles of uniform size were carefully selected and rinsed twice with a sterile saline solution. Subsequently, they were placed in a phenol-free red DMEM culture medium, with 15 vesicles allocated to each well of a 48-well cell culture plate. Various concentrations of asparagusic acid (ranging from 0 to 320 μM) were added to the vesicles, which were then incubated for 5 days. A positive control group was treated with Triton X-100 (0.1% PBS) for 5 days, with the release of PGI or γ-GGT set to 100%. After the 5-day incubation period, the culture supernatant was collected and stored at −80 °C for subsequent PGI or γ-GGT content determination. PGI and γ-GGT contents were assessed using PGI and γ-GGT assay kits, respectively, following the manufacturer’s instructions. Absorbance values at 340 nm or 415 nm were measured using an Infinite M200 PRO enzyme reader. The percentage of PGI release was calculated using the following formula: PGI release % = (OD value of asparagusic acid group − OD value of blank wells)/(OD value of Triton X-100 group − OD value of blank wells) × 100%. The EC50 values were determined using an online EC50 calculator available at https://www.aatbio.com/tools/ec50-calculator (accessed on 7 January 2024). Some of the vesicles were fixed in a 2.5% glutaraldehyde solution for subsequent scanning electron microscopy (SEM) and transmission electron microscopy (TEM) observations.

### 2.12. Effect of Asparagusic Acid on Germinal Cells

Germinal cells were isolated post-digestion of the metacestode vesicles by employing a 0.125% trypsin solution. Subsequent to cell counting, the cell density was adjusted to 1000 cells/mL. A 100 μL aliquot of cell suspension was dispensed into each well of a 96-well culture plate, followed by the addition of varying concentrations of asparagusic acid (0, 2.5, 5, 10, 20, 40, 80, 160, and 320 μM) to achieve a final volume of 200 μL. The culture plates were then enclosed within nitrogen-filled sealed bags and incubated for 5 days at 37 °C in a 5% CO_2_ incubator. Following this incubation period, 50 μL of CellTiter-Glo containing 1% Triton X-100 was added to each well, and the culture plates were further incubated for 15 min, shielded from light. Upon the complete disruption of cell aggregates, the fluorescence intensity was assessed using an Infinite M200 PRO reader. The viability of the 0 μM group was normalized to 100%. IC50 values were determined using the online IC50 calculator (https://www.aatbio.com/tools/ic50-calculator, accessed on 7 January 2024). Each concentration was subjected to four independent biological replicates.

### 2.13. Detection of Apoptosis by Flow Cytometry

The germinal cells were co-cultured with asparagusic acid for 48 h. Subsequently, the germinal cells were harvested and incubated with propidium iodide (PI) and Annexin V-FITC (BD Biosciences, Franklin Lakes, NJ, USA) for 30 min in the absence of light, followed by an apoptosis assessment using flow cytometry (ACEA Biosciences, San Diego, CA, USA).

### 2.14. Assessment of In Vitro Toxicity in Mammalian Cells

A CCK-8 assay (Elabscience) was employed to evaluate the in vitro cytotoxicity of asparagusic acid on both HFF cells and normal human hepatocytes. HFFs and normal human hepatocytes were seeded in 96-well cell culture plates at densities of 10,000 cells per well and 50,000 cells per well, respectively. Following cell adhesion, they were exposed to varying concentrations of asparagusic acid (0, 2.5, 5, 10, 20, 40, 80, 160, and 320 μM) for 48 h. Subsequently, 10 μL of CCK-8 solution was added, and the plates were incubated at 37 °C for 1 h. Absorbance at 450 nm was measured using the Infinite M200 reader (TECAN, Männedorf, Zürich, Switzerland). Six independent biological replicates were performed for each concentration.

### 2.15. Scanning Electron Microscopy (SEM) and Transmission Electron Microscopy (TEM) Analysis

The protoscoleces or metacestode vesicles were initially fixed in 2.5% glutaraldehyde buffer for 48 h. Following fixation, the samples underwent three washes with double-distilled water and were subsequently treated with 1% osmium tetroxide (SPI-CHEM, West Chester, PA, USA) for 1 h. Dehydration was then carried out using a series of ethanol concentration gradients (30%, 50%, 70%, 80%, 90%, 95%, 100%) for 15 min each. Following dehydration, the samples were briefly incubated in hexamethyldisilazane for 2 min. For an SEM analysis, the samples were air-dried in a fume hood, coated with gold via spray-plating, and examined using a JSM-IT700HR scanning electron microscope (JEOL, Tokyo, Japan). For TEM observation, the samples were embedded in Epon 812 resin and polymerized overnight at 65 °C. Subsequently, 50 nm sections were prepared and mounted onto copper grids. Finally, staining was performed with uranyl acetate and lead citrate (SPI-CHEM) prior to observation with a JEM-1400plus transmission electron microscope (JEOL).

### 2.16. In Vivo Toxicity Assessment of Asparagusic Acid

To assess the in vivo toxicity of asparagusic acid, the mice were randomly allocated into two groups (6 mice/group): the control group received daily saline gavage, while the asparagusic acid group received a daily gavage of 40 mg/kg asparagusic acid. Following 4 weeks of continuous gavage administration, the mice were euthanized under 1% pentobarbital sodium anesthesia, and blood samples were collected via the retro-orbital plexus. The collected blood samples were anticoagulated using ethylenediaminetetraacetic acid-K2 and subjected to a hematological analysis. Serum samples were obtained by incubating the blood at 37 °C for 1 h, followed by centrifugation at 5000× *g* for 10 min. Mouse liver and kidney tissues were fixed in 4% paraformaldehyde, stained with hematoxylin–eosin, and examined using a BX53 microscope (Olympus, Tokyo, Japan).

### 2.17. Evaluation of the In Vivo Effect of Asparagusic Acid on E. multilocularis Metacestodes

Metacestode vesicles were excised and intraperitoneally inoculated into the C57BL/6J mice to establish a secondary AE infection model; another seven mice served as normal controls and received intraperitoneal injections of equal volumes of normal saline. Following a 4-week infection period, the infected mice were randomly assigned to four groups (7 mice/group): the Untreated group received 0.3 mL of normal saline via gavage daily; the ABZ group received 0.3 mL of 100 mg/kg albendazole via gavage daily; the AA 10 group received 0.3 mL of 10 mg/kg asparagusic acid via gavage daily; and the AA 40 group received 0.3 mL of 40 mg/kg asparagusic acid via gavage daily. The administration of the drugs was performed daily at consistent times over a 4-week duration. Subsequently, the mice were humanely euthanized via cervical dislocation following anesthesia induced by 1% sodium pentobarbital. Abdominal cavity tissues were meticulously dissected and weighed. Metacestode tissue samples were fixed with 4% paraformaldehyde for a histopathological examination and with 2.5% glutaraldehyde for a TEM analysis.

### 2.18. Hematoxylin–Eosin (HE) Staining

Tissues were fixed in 4% paraformaldehyde, followed by dehydration and embedding in paraffin to generate 5 μm sections. These sections were then dewaxed, rehydrated, and subjected to hematoxylin–eosin staining as per the manufacturer’s protocol. The sections were observed using a BX53 microscope (Olympus).

### 2.19. Periodic Acid Schiff (PAS) Staining

Tissues were fixed in 4% paraformaldehyde, followed by dehydration and embedding in paraffin to obtain 5 μm sections. Staining was performed using a Periodic Acid Schiff (PAS) Stain Kit (Solarbio) as per the manufacturer’s protocol. The sections were visualized and imaged using a BX53 microscope (Olympus).

### 2.20. Immunohistochemical Staining

The tissue sections underwent dewaxing, antigen retrieval, and blocking with 1% BSA. Subsequently, they were incubated overnight at 4 °C with primary antibodies against Caspase3 (Proteintech, Wuhan, China) and Ki67 (Abclonal, Wuhan, China). Following this, horseradish peroxidase (HRP)-conjugated secondary antibody IgG (Abclonal) was applied and incubated for 1 h at 37 °C. Visualization of the sections was achieved by adding diaminobenzidine, followed by a 30 s restaining with hematoxylin. Finally, the sections were dehydrated and sealed with neutral gum for subsequent observation.

### 2.21. Western Blot Analysis

The protoscoleces, germinal cells, or lesion-host microenvironment tissues underwent lysis with Ripa buffer supplemented with a PMSF protease inhibitor and a phosphatase inhibitor on ice for 30 min, followed by supernatant extraction via centrifugation at 13,000× *g* at 4 °C for 5 min. The protein sample concentration was determined using a BCA protein quantification kit. Subsequently, the supernatant was diluted with 5× protein loading buffer and heated in a 95 °C water bath for 10 min, after which the protein samples were stored at −80 °C. The total protein ranging from 20 to 50 μg was subjected to polyacrylamide gel electrophoresis. Post-electrophoresis, the proteins were transferred onto a 0.2 μm PVDF membrane (Merck Millipore, Darmstadt, Germany), which was then blocked with 5% skim milk powder or 3% BSA for 1 h at room temperature. The membranes were subsequently incubated overnight at 4 °C with antibodies against Bax (1:1000, Proteintech), Bcl2 (1:1000, Proteintech), Caspase3 (1:800, Proteintech), PCNA (1:800, Proteintech), MMP2 (1:1000, Abclonal), MMP9 (1:1000, Abclonal), PI3K (1:1000, Proteintech), p-PI3K (1:800, Proteintech), AKT (1:1000, CST, Danvers, MA, USA), p-AKT (1:800, CST), and β-actin (1:1500, Abclonal). On the following day, the membranes were washed and further incubated with HRP-labelled goat anti-rabbit IgG (1:4000, Proteintech) for 1 h at room temperature. After additional washing steps, the blots were visualized using a Chemiluminescent Imaging System (SageCreation, Beijing, China). The protein bands were then quantified using Image J software, with β-actin serving as the internal control.

### 2.22. Statistical Analysis

The experimental data are presented as mean ± standard deviation. GraphPad Prism 9.0 software was utilized for graphical representation and statistical analysis. Statistical comparisons between the two groups were conducted using Student’s *t*-test. For analyses involving multiple groups, a one-way ANOVA, followed by Dunnett’s multiple comparisons test or Tukey’s multiple comparison post-test, was employed. *p* < 0.05 was deemed statistically significant.

## 3. Results

### 3.1. In Vitro Activity of Asparagusic Acid against E. multilocularis Protoscoleces

**Figure 1:**
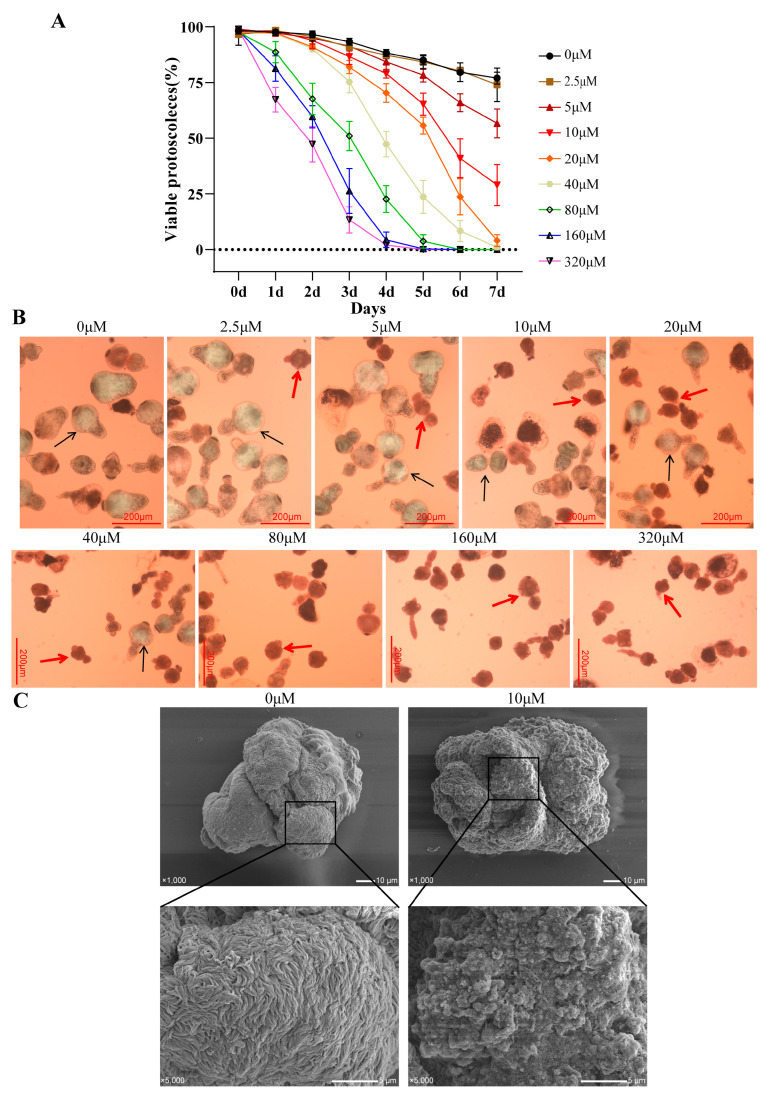
Effect of in vitro intervention with asparagusic acid on the activity of *E. multilocularis* protoscoleces. (**A**) Survival rate of protoscoleces after asparagusic acid intervention. Protoscoleces were treated with asparagusic acid for 7 days, and the survival rate of protoscoleces was evaluated after daily staining with 0.1% eosin. (**B**) Morphological observation of protoscoleces after asparagusic acid intervention. The red arrow shows the dead protoscolex, and the black arrow shows the living protoscolex. Scale bar = 200 μm. (**C**) SEM observation of protoscoleces on the 5th day after 10 μM asparagusic acid treatment.

To investigate the in vitro effect of asparagusic acid on *E. multilocularis* protoscoleces, the survival rate and the morphological changes in the protoscoleces were observed after intervention with different concentrations of asparagusic acid. A dose- and time-dependent reduction in protoscolex viability was observed over a 7-day period following treatment with 2.5–320 μM concentrations of asparagusic acid (Figure 1A). Concentrations of asparagusic acid below 5 μM demonstrated no significant impact on protoscolex survival. Following 7 days of treatment with 10 μM, 20 μM, and 40 μM concentrations of asparagusic acid, protoscolex survival rates were measured at 25.7%, 4.0%, and 1.5%, respectively. Dead protoscoleces were distinguished from their viable counterparts using the eosin rejection method, with deceased protoscoleces displaying a red stain under optical microscopy (Figure 1B). SEM revealed that while normal protoscoleces exhibited intact head hooks and microvilli structures on their surfaces, those exposed to asparagusic acid displayed disorganized microvilli structures and the detachment of head hooks (Figure 1C).

### 3.2. Effect of Asparagusic Acid on Apoptosis in Protoscoleces

**Figure 2:**
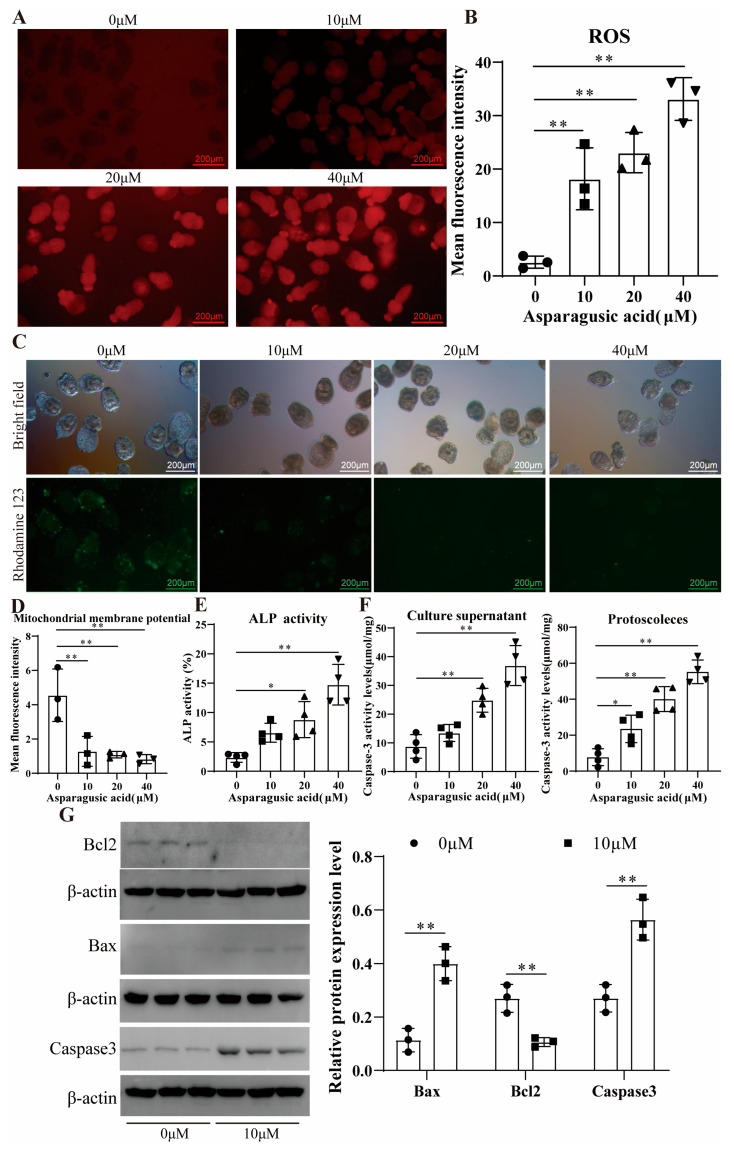
The impact of asparagusic acid on the apoptosis of *E. multilocularis* protoscoleces. (**A**,**B**) ROS levels in the protoscoleces after asparagusic acid intervention. (**C**,**D**) Levels of mitochondrial membrane potential in the protoscoleces after asparagusic acid intervention. (**E**) ALP levels in the culture supernatant of the protoscoleces after asparagusic acid intervention. (**F**) Caspase3 activity in protoscoleces and culture supernatants after asparagusic acid intervention. (**G**) Western blot detection of apoptotic protein expression levels in the protoscoleces after asparagusic acid intervention. * *p* < 0.05 or ** *p* < 0.01 compared with 0 μM group.

Normal mitochondrial function is pivotal in regulating cell proliferation and apoptosis [16]. To ascertain the involvement of reactive oxygen species (ROS) in asparagusic acid-induced apoptosis in protoscoleces, ROS levels were assessed in both protoscoleces and culture supernatants following asparagusic acid treatment (Figure 2A,B). Treatment with 10 μM, 20 μM, and 40 μM of asparagusic acid for 48 h led to escalated ROS levels in the protoscoleces. Subsequently, Rhodamine was employed to examine the impact of the asparagusic acid intervention on the mitochondrial membrane potential of protoscoleces, revealing a reduction in mitochondrial membrane potential post-intervention with asparagusic acid (Figure 2C,D). This decline in mitochondrial membrane potential serves as an indicator of early apoptosis [17,18]. ALP activity was increased in the culture supernatant of the protoscoleces after the asparagusic acid intervention (Figure 2E). Additionally, the apoptotic effect of asparagusic acid on protoscoleces was investigated. The intervention with asparagusic acid resulted in heightened Caspase3 activity in both the protoscoleces and culture supernatants (Figure 2F). A Western blot analysis revealed that the asparagusic acid intervention upregulated the expression of Bax and Caspase3 proteins while downregulating the expression of Bcl2 proteins in the protoscoleces (Figure 2G).

### 3.3. Evaluation of the In Vitro Anti-Metacestode Vesicle Effect of Asparagusic Acid

**Figure 3:**
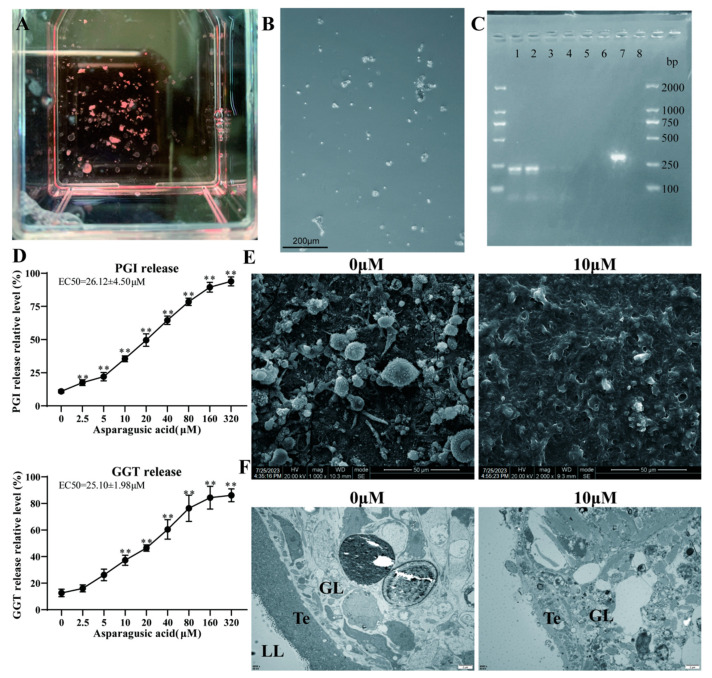
In vitro activity of asparagusic acid against metacestode vesicles. (**A**) Metacestode vesicle gross morphology. (**B**) Isolated germinal cell morphology. Scale bar = 200 μm. (**C**) Identification of cultured metacestode vesicles and germinal cells with or without host cell contamination. Lanes 1 and 5: metacestode vesicles; lanes 2 and 6: germinal cells; lanes 3 and 7: mouse liver; lanes 4 and 8: negative control. (**D**) Effect of various concentrations of asparagusic acid on PGI and γ-GGT release. 0.1% Triton X-100 was used as a positive control and set to 100% release. ** *p* < 0.01 compared with 0 μM group. (**E**) SEM observation of vesicles after 10 μM asparagusic acid intervention. (**F**) TEM observation of vesicles after 10 μM asparagusic acid intervention. GL: germinal layer; Te: tegument; LL: laminated layer.

We successfully cultured metacestode vesicles (Figure 3A) and digested them using trypsin to obtain germinal cells (Figure 3B). An RT-PCR analysis showed no mouse GAPDH amplification in the metacestode vesicles or germinal cells, demonstrating no host cell contamination (Figure 3C). The release level of PGI and γ-GGT in the culture medium was assessed following exposure to various concentrations of asparagusic acid targeting the metacestode vesicles. A direct correlation was observed between the concentration of asparagusic acid and the PGI and γ-GGT contents in the culture supernatant (Figure 3D). The EC50 values for PGI and γ-GGT were 26.12 ± 4.50 μM and 25.10 ± 1.98 μM, respectively. Subsequently, SEM and TEM were employed to further investigate the microstructural alterations induced by the 10 μM asparagusic acid intervention. An SEM examination of the inner vesicle surface revealed the presence of small vesicles adhering to the germinal layer in the non-intervention group, while such structures were notably absent in the group subjected to the asparagusic acid intervention (Figure 3E). A TEM analysis of the vesicle wall exhibited characteristic features, including the germinal layer, tegument, and laminated layer in the non-intervention group, whereas after the asparagusic acid intervention, necrosis, and apoptotic bodies were observed within the cells of the germinal layer (Figure 3F).

### 3.4. Effect of Asparagusic Acid on the Viability of E. multilocularis Germinal Cells

**Figure 4:**
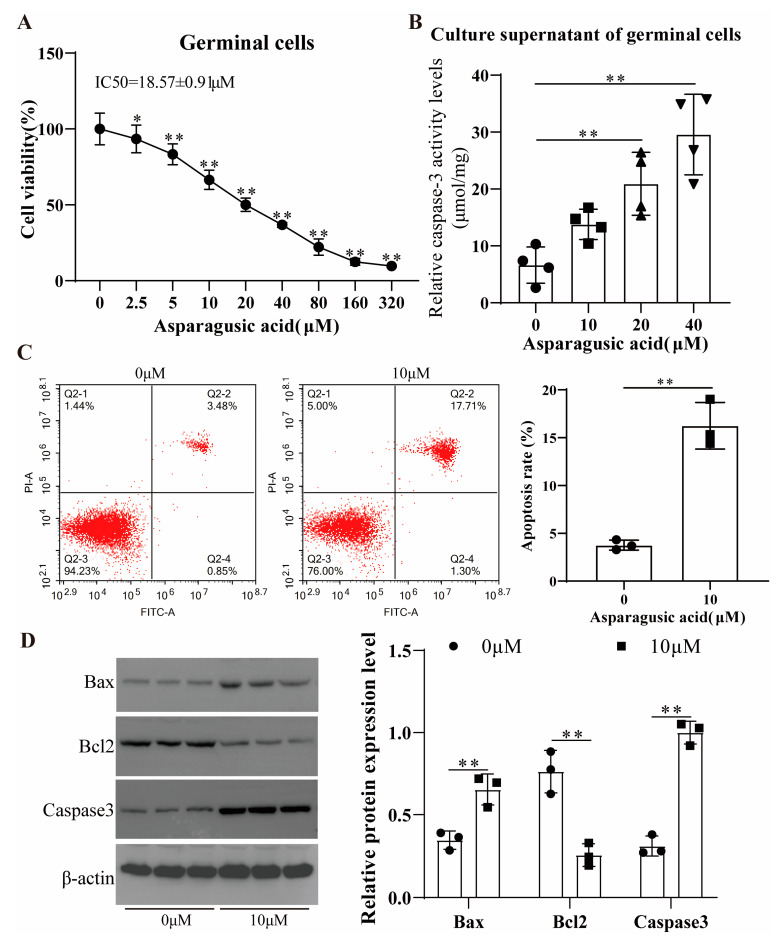
Cytotoxicity of asparagusic acid on *E. multilocularis* germinal cells. (**A**) Asparagusic acid inhibited the viability of germinal cells. (**B**) Caspase3 activity level in germinal cell culture supernatant after asparagusic acid intervention. (**C**) Annexin-V+ PI staining was used to detect apoptosis of germinal cells. (**D**) Western blot was used to detect the expression level of apoptosis-related proteins. * *p* < 0.05 or ** *p* < 0.01 compared with 0 μM group.

Germinal cells were isolated from the metacestode vesicles cultured in vitro through trypsin digestion. The impact of asparagusic acid on germinal cell activity was assessed using a CellTiter-Glo assay. Asparagusic acid exhibited a significant inhibitory effect on the viability of the germinal cells (Figure 4A). The IC50 value of asparagusic acid against the germinal cells was measured at 18.57 ± 0.91 μM. Treatment with 2.5 μM asparagusic acid resulted in a notable decline in germinal cell viability. Upon intervention with 20 μM asparagusic acid, the inhibition rate of the germinal cells exceeded 50%. Furthermore, exposure to asparagusic acid induced an increase in Caspase3 activity within the cell culture supernatants of the germinal cells (Figure 4B). A flow cytometric analysis demonstrated that asparagusic acid facilitated the apoptosis of the germinal cells (Figure 4C). A subsequent Western blot analysis revealed alterations in the expression levels of apoptosis-associated proteins following asparagusic acid treatment, with an increased expression of Bax and Caspase3 and a decreased expression of Bcl2 (Figure 4D).

### 3.5. In Vitro Toxicity Evaluation of Asparagusic Acid on Human Normal Hepatocytes and HFFs

**Figure 5:**
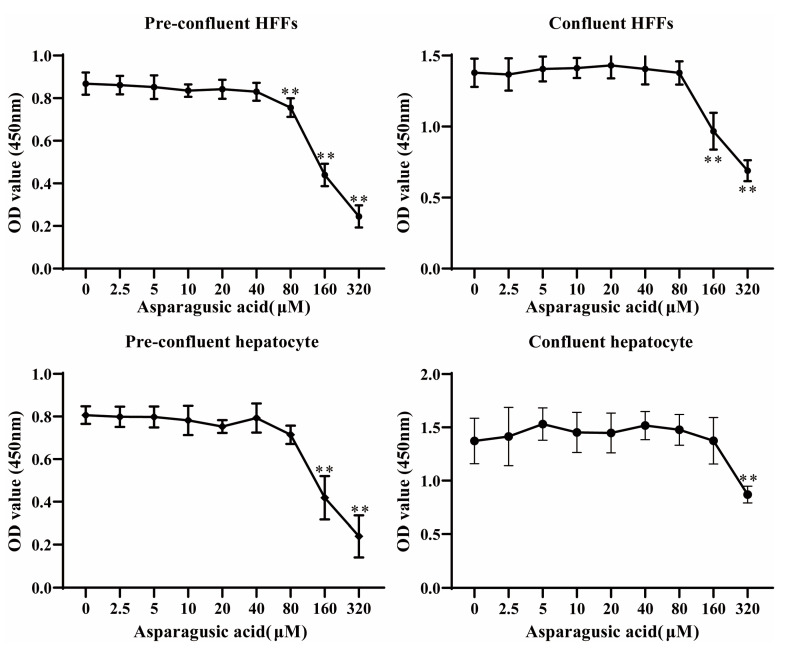
Cytotoxicity of asparagusic acid on mammalian cells. The activity of various concentrations of asparagusic acid on pre-confluent or confluent human normal hepatocytes and HFFs was assessed using a CCK-8 assay. ** *p* < 0.01 compared with 0 μM group.

The cytotoxicity of asparagusic acid was assessed using the CCK-8 method on pre-confluent or confluent human normal hepatocytes and HFF cells (Figure 5). The CCK-8 results revealed that concentrations of asparagusic acid ranging from 2.5 μM to 80 μM exhibited no discernible impact on either pre-confluent or confluent normal human hepatocytes and HFFs. However, concentrations of asparagusic acid exceeding 160 μM exhibited a pronounced inhibitory effect on the proliferation of pre-confluent human normal hepatocytes, while concentrations exceeding 320 μM had a similar effect on confluent human normal hepatocytes. Moreover, concentrations of asparagusic acid above 80 μM exhibited a significant inhibitory effect on the growth of pre-confluent HFFs, whereas concentrations above 160 μM had a comparable effect on confluent HFFs. Notably, the toxicity of asparagusic acid towards mammalian cells was notably lower than its effects on the protoscoleces, metacestode vesicles, and germinal cells.

### 3.6. Toxicity Evaluation of Asparagusic Acid in Mice

**Figure 6:**
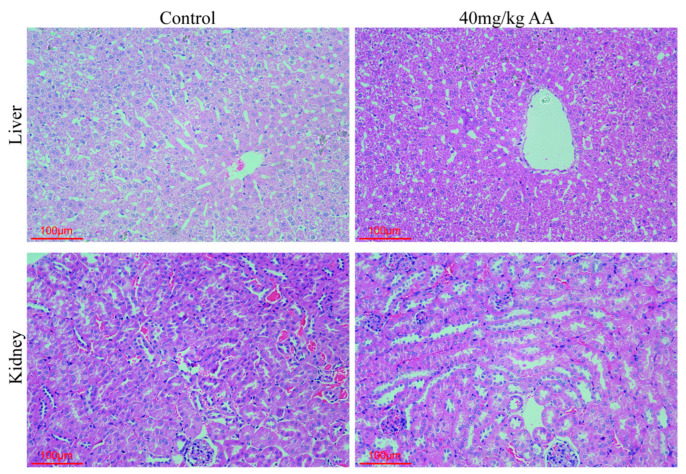
Pathological examination of liver and kidney in mice after asparagusic acid treatment.

The toxicity of asparagusic acid in vivo was evaluated in normal mice. Specifically, normal C57BL/6J mice were subjected to a 4-week treatment with 40 mg/kg asparagusic acid. Blood samples were collected for the assessment of white blood cell counts, red blood cell counts, hemoglobin levels, and platelet counts, while serum samples were analyzed for the levels of ALT, AST, ALP, TP, and LDH. Notably, no significant differences were observed in the aforementioned hematological and biochemical parameters between the Control group and the group treated with 40 mg/kg asparagusic acid (Table 1). Furthermore, a histopathological examination of liver and kidney tissues stained with HE revealed no discernible histopathological alterations or injuries following the intervention with asparagusic acid (Figure 6).

### 3.7. In Vivo Effect of Asparagusic Acid against E. multilocularis Metacestodes

**Figure 7:**
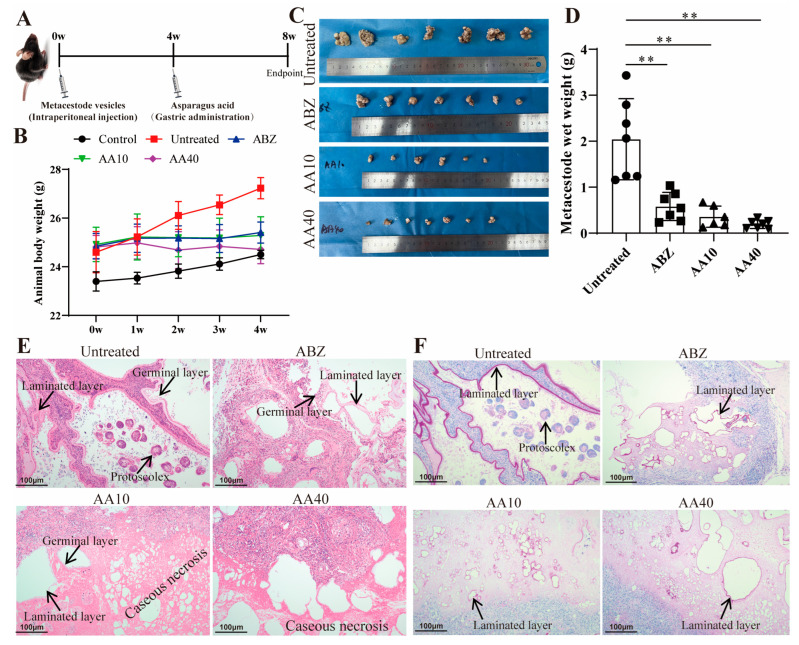
In vivo activity of asparagusic acid against *E. multilocularis* metacestodes. After C57BL/6J mice were intraperitoneally injected with *E. multilocularis* vesicle tissue for 4 weeks, the mice were given asparagusic acid by gavage for 4 weeks. Albendazole at a dose of 100 mg/kg served as the positive control. (**A**) Schematic diagram of the effect of asparagic acid on mice with secondary AE infection. (**B**) Body weight change in mice. (**C**) Images of metacestodes resected from different treatment groups. ** *p* < 0.01 compared with the Untreated group. (**D**) Metacestode weight in different treatment groups. (**E**) Metacestode lesions were observed via HE staining. Scale bar = 100 μm. (**F**) Laminated layer PAS staining. Scale bar = 100 μm.

The impact of asparagusic acid treatment on parasite load was investigated in a murine model of secondary infection with *E. multilocularis* (Figure 7A). No significant increase in body weight was observed in the mice after the intervention with albendazole and asparagusic acid (Figure 7B). Following four weeks of asparagusic acid administration, a significant reduction in metacestode wet weight was observed at doses of 10 mg/kg and 40 mg/kg compared to the Untreated group (Figure 7C,D). Similarly, albendazole demonstrated efficacy in reducing metacestode wet weight (Figure 7C,D). Although asparagusic acid appeared to exhibit greater efficacy post-treatment than ABZ, no statistically significant difference was detected in the wet weight of their lesions (Figure 7D). Notably, asparagusic acid treatment exhibited no evidence of concentration-dependent tolerance. A histological examination of lesion tissue following HE staining revealed distinct protoscoleces, laminated layers, and germinal cells in the Untreated group (Figure 7E). Conversely, after asparagusic acid treatment, conspicuous caseous necrosis and residual laminated layers were evident (Figure 7E). Furthermore, laminated layers were still detectable following periodic acid-Schiff (PAS) staining in all treatment groups (Figure 7F).

### 3.8. Ultrastructural Changes in the Lesion–Host Microenvironment after Asparagusic Acid Treatment

**Figure 8:**
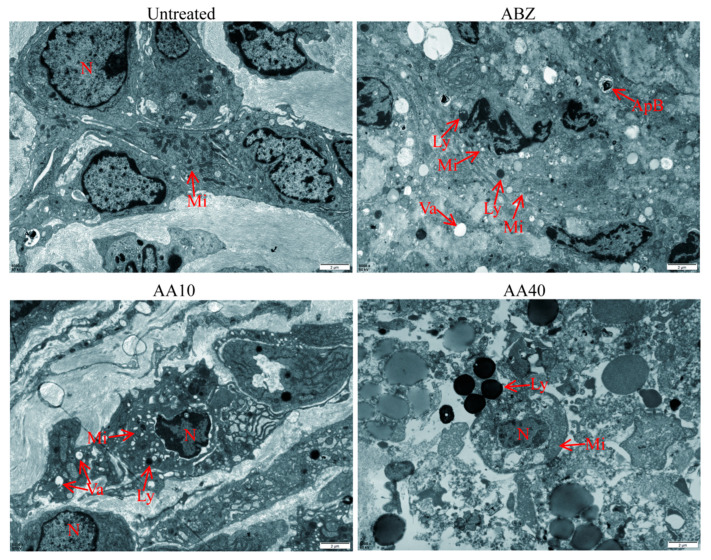
Ultrastructural observation of the lesion–host microenvironment. Scale bar = 2 μm. N: nucleus; Mi: mitochondria; Va: vacuole; Ly: lysosome; ApB: apoptotic body.

The ultrastructure of the focus-host microenvironment was observed using TEM (Figure 8). The fibroblasts in the Untreated group exhibited morphological integrity, displaying well-defined organelle structures within the cytoplasm that were devoid of any discernible lesions. The fibroblasts in the ABZ group displayed necrotic features characterized by indistinct cell boundaries, a disorganized cytoplasmic architecture, mild mitochondrial swelling, and slight chromatin aggregation. The fibroblasts in the AA10 group maintained morphological integrity, showcasing cytoplasmic mitochondrial swelling and a sparse presence of lysosomes. Conversely, the fibroblasts in the AA40 group exhibited severe necrosis, evidenced by pronounced structural disintegration, resulting in a loss of cellular integrity, with only remnants of cellular structures discernible.

### 3.9. Effect of Asparagusic Acid on Apoptosis in the Lesion–Host Microenvironment

**Figure 9:**
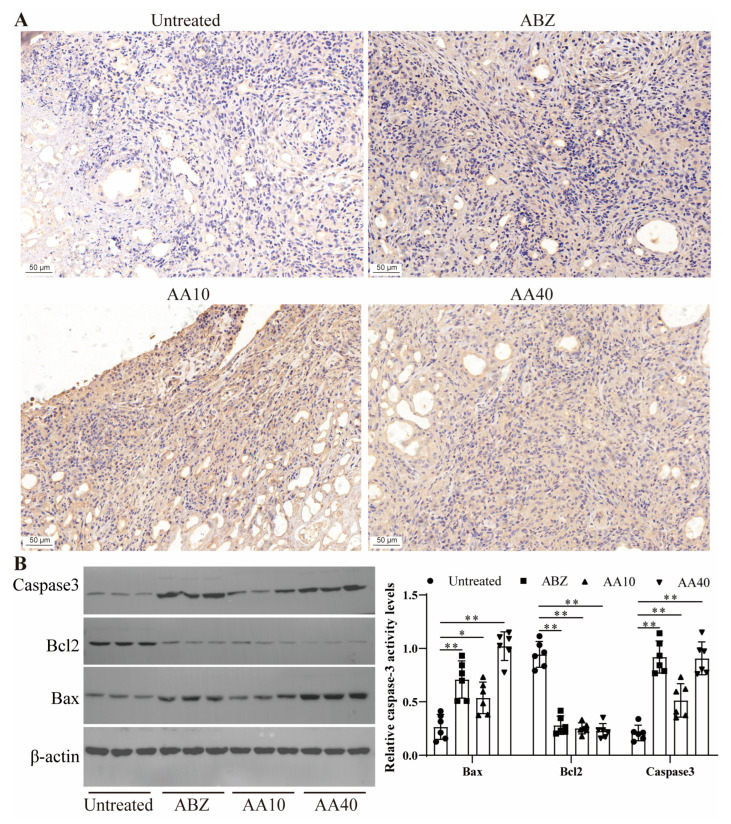
Effect of asparagusic acid on apoptosis in the lesion–host microenvironment. (**A**) Immunohistochemical detection of Caspase3 protein expression. Scale bar = 50 μm. (**B**) Western blot detection of apoptosis-related protein expression and quantitative analysis. * *p* < 0.05 or ** *p* < 0.01 compared with Untreated group.

Following immunohistochemical staining, observations were made regarding the expression level of Caspase3 in the lesion–host microenvironment (Figure 9A). The findings suggest that the treatment with asparagusic acid led to an augmentation in Caspase3 protein expression within the lesion-host microenvironment. A subsequent Western blot analysis revealed an increase in the levels of Bax and Caspase3 proteins in the lesion–host microenvironment, coupled with a reduction in the expression level of the Bcl2 protein subsequent to the asparagusic acid intervention (Figure 9B).

### 3.10. Effect of Asparagusic Acid on Proliferation of Lesion–Host Microenvironment

**Figure 10:**
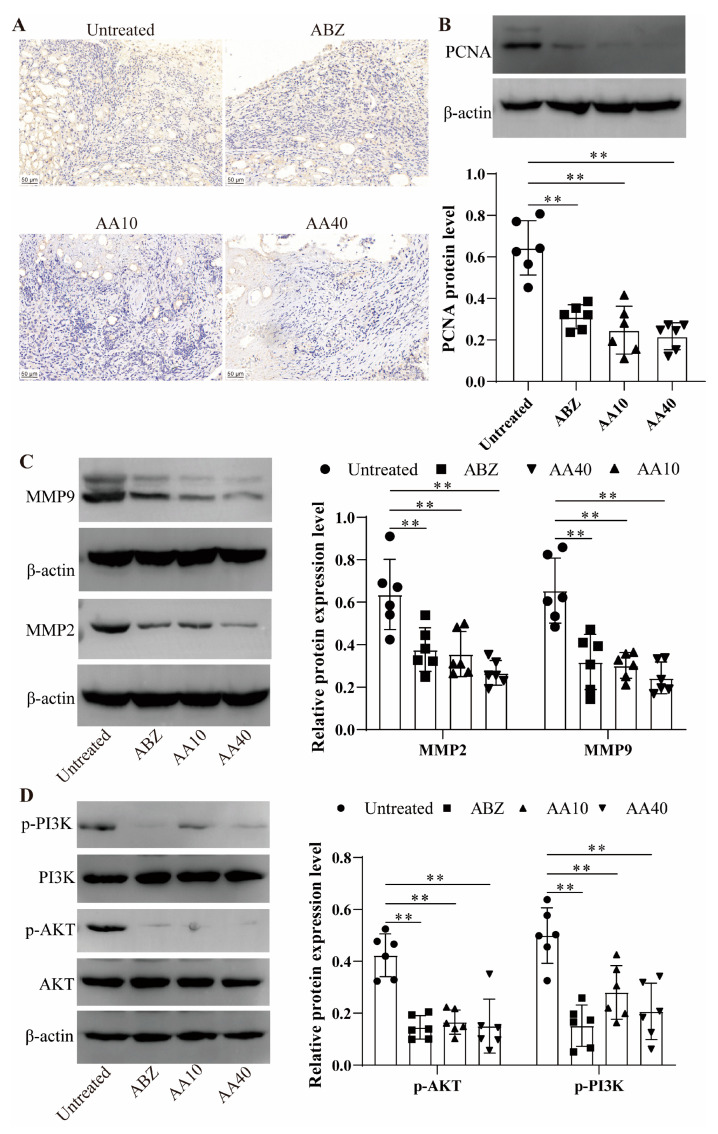
Effect of asparagusic acid on tissue proliferation and PI3K/AKT signaling pathway in the lesion–host microenvironment. (**A**) The expression of the Ki67 protein was detected via immunohistochemistry. Scale bar = 50 μm. (**B**) A western blot was used to detect the expression of PCNA protein. (**C**) Western blot was used to detect the expression levels of MMP2 and MMP9 proteins. (**D**) Western blot was used to detect the expression levels of p-PI3K and p-AKT proteins. ** *p* < 0.01 compared with the Untreated group.

Caspase3 expression levels were assessed in the lesion-host microenvironment via immunohistochemical staining. The findings demonstrated a decrease in Ki67 expression within the lesion–host microenvironment subsequent to the asparagusic acid treatment (Figure 10A). A Western blot analysis further confirmed a decline in PCNA protein expression in the lesion–host microenvironmental tissues post-asparagusic acid intervention (Figure 10B). Additionally, the protein expression levels of MMP2 and MMP9 were evaluated in the same tissues, revealing a decrease in MMP2 and MMP9 expression levels following treatment with asparagusic acid (Figure 10C).

### 3.11. Effect of Asparagusic Acid on PI3K/AKT Signaling Pathway in Lesion–Host Microenvironment

The pivotal involvement of the PI3K/AKT signaling pathway in the modulation of apoptosis or cell proliferation underscores its significance. Consequently, the impact of the asparagusic acid treatment on the PI3K/AKT signaling pathway within the lesion–host microenvironment was subjected to detailed scrutiny (Figure 10D). Western blot analysis revealed notable reductions in the expression levels of p-PI3K and p-AKT in both the ABZ group and the asparagusic acid group, as compared to the Untreated group.

## 4. Discussion

**Figure 11:**
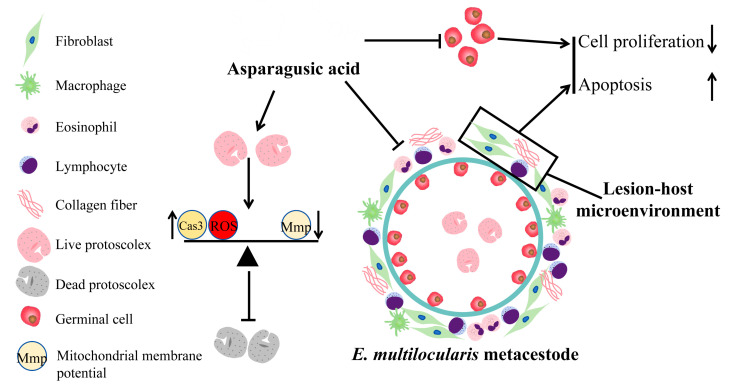
Schematic diagram of asparagusic acid against *E. multilocularis*.

Alveolar echinococcosis is a zoonotic disease caused by *E. multilocularis* larvae on intermediate hosts or definitive hosts [19]. Untreated cases entail a considerable mortality rate, rendering AE among the most perilous food-borne parasites globally [20]. Albendazole, while effective, manifests parasitostatic rather than parasitocidal properties, necessitating prolonged administration [21]. Hence, there is a pressing need for novel therapeutic agents or lead compounds. This investigation revealed that asparagusic acid exhibits protoscolicidal activity, impedes germinal cell function, and demonstrates promising anti-echinococcosis efficacy in vivo (Figure 11). These findings posit asparagusic acid as a potential candidate for the development of anti-*E. multilocularis* pharmaceuticals.

Herbal medicines play a pivotal role in drug discovery and development, representing a rich source with diverse medicinal potential [22]. Natural products have long been acknowledged as valuable reservoirs for novel drug development [23]. In contrast to chemically synthesized compounds, natural products carry a minimal risk of adverse reactions [24]. To date, numerous Chinese herbal extracts have demonstrated efficacy in protoscolex eradication, and they exhibit promising immunomodulatory properties [25]. Maintaining low levels of reactive oxygen species (ROS) within cells is crucial for regulating cell survival, as excessive ROS can trigger oxidative stress-induced cellular damage and apoptosis [26]. This study revealed that asparagusic acid exhibits concentration- and time-dependent parasiticidal activity against protoscoleces, accompanied by an increase in ROS levels and a reduction in mitochondrial membrane potential. An increase in ROS levels, the subsequent opening of mitochondrial permeability transition pores, and a decrease in mitochondrial membrane potential constitute common mechanisms through which antitumor agents induce cell death across various cancer types [27,28]. In a previous study, it was found that treatment with asparagus extracts significantly enhanced apoptosis and reduced the proliferation of myeloid-derived suppressor cells (MDSCs) via the intrinsic pathway [29]. Mitochondria not only serve as the cellular powerhouse but also play a crucial role in signaling transduction and apoptotic regulation [30]. Following the intervention with asparagusic acid, Caspase3 activity increased in the protoscoleces, along with the levels of alkaline phosphatase (ALP) and Caspase3 in the culture supernatant. Additionally, the asparagusic acid intervention upregulated the expression of Bax and Caspase3 proteins while downregulating Bcl2 protein expression. ALP participates in the regulation of protein phosphorylation, cell growth, apoptosis, and cell migration [31]. These findings suggest that asparagusic acid may expedite protoscolex apoptosis by upregulating Caspase3.

Enhanced AE chemotherapy regimens are currently under active investigation as potential substitutes for the benzimidazoles currently in use. A streamlined method for the efficient and reliable isolation and cultivation of *E. multilocularis* vesicles and mucous layer cells facilitates the screening of anti-echinococcosis drugs [32]. The germinal layer of *E. multilocularis* comprises diverse cell types, including muscle cells, glycogen storage cells, and undifferentiated cells [33]. Targeting *E. multilocularis* stem cells is essential to achieve parasiticidal effects [34]. In this investigation, the intervention with asparagusic acid resulted in elevated PGI and γ-GGT release in vesicle culture supernatants, inducing the structural disruption of the germinal layer. Asparagusic acid suppressed the proliferative activity and promoted the apoptosis of germinal cells. GGT release from the tegument preceded vesicle structural damage, serving as a sensitive viability indicator of *E. multilocularis* metacestode [35]. These findings underscore the ability of asparagusic acid to induce apoptosis and impede the proliferation of germinal cells, thereby exerting an anti-*E. multilocularis* effect.

Drug toxicity stands as a significant constraint on drug utilization in the course of drug development. Previous investigations have demonstrated the nearly negligible harm inflicted upon humans by asparagus upon ingestion [12]. In the present study, asparagusic acid concentrations below 80 μM exhibited no discernible deleterious impacts on pre-confluent and confluent HFF cells or on normal human hepatocytes. The cytotoxicity of asparagusic acid on mammalian cells was notably lower than its impact on the protoscoleces, metacestode vesicles, and germinal cells.

The inhibition rate of lesion growth serves as a visual measure of drug efficacy against *E. multilocularis*. The current findings demonstrate a significant inhibition of metacestode growth in vivo by asparagusic acid, indicating its potential anti-*E. multilocularis* effect in vivo. The asparagusic acid intervention disrupted the germinal layer structure within the lesions, resulting in a more pronounced residual laminated layer structure. Furthermore, the intervention with asparagusic acid led to an increase in vacuolation and mitochondrial swelling in cells within the lesion–host microenvironment. Mitochondria play a crucial role in cellular energy production via the promotion of carbohydrate metabolism. The swelling and fragmentation of mitochondria suggest compromised mitochondrial function, potentially leading to apoptosis or cell death [36]. The activation of Caspase3 serves as a biochemical marker for both early and late stages of apoptosis, and assessing the relative expression levels of Caspase3 in cells or tissues is crucial for elucidating apoptosis occurrence [37]. Accordingly, the expressions of Bax, Bcl2, and Caspase3 were examined in perilesional tissues to investigate the impact of asparagusic acid on apoptosis. Following the asparagusic acid treatment, there was a notable increase in Caspase3 protein expression in the perilesional tissues. Moreover, elevated levels of Bax and Caspase3 proteins, alongside reduced levels of the Bcl2 protein, were observed in the perilesional tissues post-asparagusic acid treatment. These findings suggest that asparagusic acid induces apoptosis in the surrounding tissues of lesions, thereby exerting an anti-parasitic effect.

Asparagusic acid manifests cytostatic and antitumor effects, which are attributed to its thiol/disulfide bonds [12]. Following the asparagusic acid treatment, a reduction in Ki67 and PCNA protein expression was observed in the perilesional tissues, implying the inhibition of cell proliferation in the vicinity of the lesions. Matrix metalloproteinases (MMPs) are pivotal in extracellular matrix degradation, fostering conditions conducive to tumor metastasis, and they are typically upregulated in tumor tissues [38]. MMPs facilitate cell proliferation and migration, and they exert an influence on apoptosis, angiogenesis, tissue regeneration, and the immune response [39]. In this study, the expression of MMP-2 and MMP-9 decreased in the lesion–host microenvironment after the asparagusic acid treatment, which indicates that the inhibition of lesion growth by asparagusic acid may be associated with the attenuation of the function of MMPs.

The PI3k-Akt signaling pathway orchestrates a spectrum of biological processes (e.g., cell proliferation, apoptosis, invasion, metastasis, and angiogenesis) and holds significant relevance to tumorigenesis [40]. *E. multilocularis* infection induces alterations in glucose metabolism, macrophage polarization, and the PI3K/Akt/mTOR signaling cascade [41]. Previous investigations have delineated how *E. multilocularis* microcyst fluid can stimulate fibroblast MMP2 and MMP9 expression within lesions via PI3K/Akt signaling pathway activation [42]. Our findings illustrate that treatment with asparagusic acid correlates with diminished levels of p-PI3K and p-AKT protein expression in the microenvironmental tissue of the lesion–host interface. Hence, the anti-echinococcosis potential of asparagusic acid may stem from its regulatory influence on the PI3K/AKT signaling pathway within the lesion–host microenvironment, impacting cellular proliferation and apoptosis.

## 5. Conclusions

In conclusion, the data presented herein demonstrate the efficacy of asparagusic acid in inducing mortality in *E. multilocularis* protoscoleces and suppressing the functionality of metacestode vesicles and germinal cells. Asparagusic acid induces elevated levels of ROS and mitochondrial membrane potential in protoscoleces, thereby facilitating apoptosis in both protoscoleces and germinal cells.

## Figures and Tables

**Table 1:** Serum biochemical findings and blood cell analysis in C57BL/6J mice treated with asparagusic acid for 4 weeks (*n* = 6).

C57BL/6J Mice	Control	AA40	*p*
Alanine aminotransferase (U/L)	64.05 ± 10.78	62.32 ± 6.39	0.7417
Aspartate aminotransferase (U/L)	198.42 ± 50.16	193.93 ± 28.85	0.8533
Alkaline phosphatase (U/L)	87.83 ± 6.05	84.82 ± 6.38	0.4204
Total protein (g/L)	59.20 ± 2.98	61.53 ± 4.52	0.3164
Albumin (g/L)	25.52 ± 3.23	31.00 ± 2.46	0.0079
Lactate dehydrogenase (U/L)	248.92 ± 72.04	211.43 ± 32.90	0.2733
White blood cell (10^9^/L)	4.56 ± 0.92	4.14 ± 1.57	0.5856
Red blood cell (10^9^/L)	9.23 ± 1.07	9.77 ± 0.57	0.2979
Hemoglobin (g/L)	149.67 ± 13.92	151.67 ± 7.37	0.7622
Platelet (10^9^/L)	769.17 ± 253.92	866.33 ± 101.04	0.4042

## Data Availability

The data used in the study are available from the corresponding author upon reasonable request.
